# The BRD4 inhibitor JQ1 augments the antitumor efficacy of abemaciclib in preclinical models of gastric carcinoma

**DOI:** 10.1186/s13046-023-02615-2

**Published:** 2023-02-09

**Authors:** Mei Feng, Hao Xu, Wenyuan Zhou, Yisheng Pan

**Affiliations:** 1 Division of General Surgery, Peking University First Hospital, Peking University, No. 8 Xi Shiku Street, Beijing, 100034 China; 2 NMPA Key Laboratory for Research and Evaluation of Radiopharmaceuticals (National Medical Products Administration), Department of Nuclear Medicine, Peking University Cancer Hospital & Institute, Beijing, 100142 China

**Keywords:** Gastric cancer, Synthetic lethality, CDK4/6 inhibitors, BRD4, Drug combination

## Abstract

**Background:**

Advanced gastric cancer (GC) is a lethal malignancy, harboring recurrent alterations in cell cycle pathway, especially the CDKN2A-CDK4/CDK6/CCND1 axis. However, monotherapy of CDK4/6 inhibitors has shown limited antitumor effects for GC, and combination treatments were urgently needed for CDK4/6 inhibitors.

**Methods:**

Here, we performed a comprehensive analysis, including drug screening, pan-cancer genomic dependency analysis, and epigenetic sequencing to identify the candidate combination with CDK4/6 inhibitors. Mechanisms were investigated by bulk RNA-sequencing and experimental validation was conducted on diverse in vitro or in vivo preclinical GC models.

**Results:**

We found that the BRD4 inhibitor JQ1 augments the antitumor efficacy of the CDK4/6 inhibitor abemaciclib (ABE). Diverse in vitro and in vivo preclinical GC models are examined and synergistic benefits from the combination therapy are obtained consistently. Mechanistically, the combination of ABE and JQ1 enhances the cell cycle arrest of GC cells and induces unique characteristics of cellular senescence through the induction of DNA damage, which is revealed by transcriptomic profiling and further validated by substantial in vitro and in vivo GC models.

**Conclusion:**

This study thus proposes a candidate combination therapy of ABE and JQ1 to improve the therapeutic efficacy and worth further investigation in clinical trials for GC.

**Supplementary Information:**

The online version contains supplementary material available at 10.1186/s13046-023-02615-2.

## Background

Gastric cancer (GC) is a life-threatening disease and the fourth leading cause of death worldwide [1]. To date, surgical resection is considered the main curative treatment option and chemotherapy is the most common treatment for GC [2]. Standard of first-line therapy for GC typically consists of 5-FU and a platinum-based agent including SOX (S-1 + oxaliplatin), XELOX (oxaliolatin + capecitabine), FOLFOX (leucovorin + 5-FU + oxaliplatin) and FLOT (5-FU + leucovorin + oxaliplatin + docetaxel) [3]. To date, two targeted therapies have been permitted to use in the clinic: trastuzumab (targeting HER2) and ramucirumab (targeting VEGF) [2, 4, 5]. However, these therapies have limited therapeutic effects, hence, new therapies are urgently needed for the treatment of GC [6, 7].

Cell cycle control is often disrupted in GC, making it an attractive therapeutic target [8]. Notably, CDKN2A-CDK4/CDK6/CCND1 axis was affected recurrently: CDKN2A silencing was found in 35% of GC patients, CDK6 amplification was observed in 9% of GC patients and CCND1 amplification in 7% [9, 10]. These alterations accelerate tumor cell transition from G1 to S phase and result in uncontrolled cell proliferation [11–14]. Three CDK4/6 inhibitors (abemaciclib, ABE; ribociclib, RIB; palbociclib, PAL) have been approved for the treatment of hormone-receptor-positive, HER2-negative, advanced breast cancer and more CDK4/6 inhibitors have entered clinical trials for the treatment of various cancer types [15–19]. Nevertheless, few preclinical investigations and clinical trials of CDK4/6 inhibitors have been performed on GC patients, and thus a comprehensive inspection of the pharmacological effect of CDK4/6 inhibitors in GC remains lacking.

Epigenetic drug discovery field has witnessed significant advancement in recent years [20]. Extensive preclinical studies with drugs targeting the epigenetic proteins, including writers (DNA methyltransferase DNMTs, histone methyltransferase HMTs), readers (bromodomain and extraterminal BETs) and erasers (histone deacetylase HDACs), are being explored in pursuit of tumor inhibition. To date, FDA has approved seven agents – DNMT inhibitors (Azacitidine, Decitabine), EZH2 inhibitor (Tazemetostat), HDAC inhibitors (Vorinostat, Romidepsin, Belinostat, and Panobinostat) – for the treatment of diverse malignancies [20–22], several of which exhibited attractive antitumor effects in combination with CDK4/6 inhibitors [23–25]. For instance, FK228, an epigenetic eraser inhibitor (histone deacetylase inhibitor; HDACi) exhibiting anti-tumor effects against several types of solid tumors, has been shown to restrict cell proliferation and induce apoptosis in GC cells and suppress tumor growth in GC mouse models [26]. Meanwhile, taking advantage of CRISPR-Cas9 screen technique, Goodwin, C. M., et al. recently also reported that HDAC loss could enhance the growth inhibitory activity of CDK4/6 inhibitors [27]. Hence, it would be interesting to examine whether a combination of epigenetic drug with CDK4/6 inhibitor could augment the antitumor efficacy and provide a more potent treatment for patients with advanced GC. However, comprehensive analysis of promising combination epigenetic candidates for CDK4/6 inhibitors in GC are still lacking.

In this study, we performed drug screening, pan-cancer genomic dependency analysis, epigenetic sequencing, bulk RNA-sequencing, and experimental validation on diverse in vitro and in vivo preclinical GC models. We observed pharmacological inhibition of ABE in GC cells. Moreover, we screened out BRD4 inhibitor JQ1 as a promising combination candidate for ABE. The doublet treatment of ABE and JQ1 showed encouraging antitumor effects in various GC cell lines as well as the in vivo CDX model of GC. We further demonstrated that JQ1 enhanced the cell cycle arrest of GC cells induced by ABE and the combination stimulated unique characteristics of cellular senescence triggered by DNA damage, which was not observed in either monotherapy. Collectively, our findings propose a candidate combination therapy of ABE and JQ1 to improve the therapeutic efficacy and combat cancer progression of GC.

## Materials and methods

### Cell lines, drugs, antibodies and assay reagents

Thirteen human gastric cancer cell lines were used in this study (Table S1) and all cultures maintained at 37 °C in the presence of 5% CO2. SNU5 (IMDM + 20% FBS); MKN45, NCI-N87, SNU16, HGC27, AGS, NUGC3, SNU1 and NUGC4 (RPMI 1640 + 10% FBS); KATOIII (IMDM + 20% FBS); MGC803 and BGC823 (DMEM + 10% FBS). Abemaciclib (#S5716) and JQ1(#S7110) were obtained from SelleckChem LLC (Houston,TX). Ki-67 Rabbit mAb (#15,580) was purchased from Abcam. HRP conjugated Goat Anti-Rabbit IgG (H + L) secondary antibody (Servicebio Cat#GB23303, RRID:AB_2811189) was purchased from Servicebio. Cell Titer-Glo® (#G7570) was obtained from Promega. Crystal violet was obtained from Sigma. Antibodies against p21 (#2947T), p53 (#2524S), Phospho-Rb (Ser807/811) (#8516T), 53BP1 (#88,439) were purchased from Cell Signaling Technology (Danvers, MA). Anti- γH2AX (Ser139) (#05-636) was purchased from Millipore.

### Dose-dependent assay

Cells were plated in 96-well plates at optimized cell density and dosed 24 h later (post-adherence). Relative luminescent signal was measured with CellTiter-Glo (CTG) after 72 h of drug exposure using a SYNERGY H1 microplate reader (BioTek, VT USA), and relative cell numbers (expressed as % viability) were calculated. Ten serially-diluted doses of abemaciclib, each in technical quadruplicates per biological replicate were evaluated. Prism version 7.03 (Prism Software Corporation, CA USA) was used to calculate concentrations corresponding to 50% viability (IC50).

### Drug synergy analysis

Cells were plated in 384-well plates (Nunc, NC USA) at optimized cell density and dosed 24 h later (post-adherence). Relative luminescent signal was measured with CellTiter-Glo (CTG) after 72 h of drug exposure using a SYNERGY H1 microplate reader (BioTek, VT USA), and relative cell numbers (expressed as % viability) were calculated. We evaluated 5 doses of JQ1 were combined with 5 doses of abemaciclib (24 dose combinations) in quadruplicate, Synergism was analyzed and visualized with SynergyFinder, (RRID:SCR_019318) using the Bliss model.

### Histopathology, immunohistochemistry of paraffin samples

Murine stomach tissue was fixed overnight in 4% formalin and then changed into 70% ETOH for preservation. The samples were fixed in paraffin. Twenty series of 5 mM sections were obtained with a sliding microtome. For Ki67, pRB, 53BP1, γ-H2AX, p21 and p53 staining of deparaffinized and hydrated sections, a heat-induced antigen retrieval method was performed for 10 min using sodium citrate buffer. Liquid DAB plus substrate reagent was used to perform direct chromogenic visualization.

### Immunofluorescence analysis

For immunofluorescence detection, cells were seeded on the round glass of 6-well plate at a density of 3 × 10^5^ cells/well. After different treatments, the cells were fixed with 4% paraformaldehyde in PBS for 20 min. Then cells were permeabilized with 0.2% Triton X 100 at room temperature for 5 min. Then the cells were sealed in 1% BSA/PBS at room temperature for 1 h. γH2AX, p53 and p21 were detected using the corresponding antibodies. Cell chromatin was stained with DAPI. Images were collected by Pannoramic DESK, P-MIDI, P250 (3D HISTECH). Qupath software was used for the quantification. Cells with more than 10 lesions were considered positive. For each condition, at least three microscopic fields were quantified.

### Colony formation assay

One thousand cells were seeded in 6-well plates, allowed to attach overnight and exposed to Abemaciclib (100nM), JQ1(100nM) or Abemaciclib (100nM) + JQ1 (100 nM) for 14 days. The medium was changed every three days. After 14 days of treatment, cells were washed with 1 × PBS and fixed with 4% paraformaldehyde (Sigma-Aldrich) for 20 min at room temperature. After washing with 1 × PBS for twice, cells were stained with crystal violet (sigma) (0.5 g crystal violet in 80 ml H2O with 20 ml methanol) for 30 min at room temperature [28]. Cells were washed with distilled water gently to remove the remaining crystal violet.

### In vivo experiments

Balb/c-Nu Mice were housed in a 12-hour light/dark cycle with access to food and water with no fasting. HGC27 cells were implanted s.c. into mice as described [29]. Tumor dimensions were monitored every three days by manual palpation and digital caliper. For the CDX models, when tumors reached the volume of 100 mm3, mice were randomized according to tumor volume into four groups (8 mice per group): (1) treated with Vehicle; (2) treated with abemaciclib (50 mg/kg orally by gavage, daily); (3) administered with JQ1 (35 mg/kg, i.p.injection, daily);and (4) treated with a combination of JQ1 (35 mg/kg, i.p. daily) and abemaciclib (50 mg/kg orally by gavage, daily). Treatment study continued for 27 days. Tumor dimension and volume (1/2 x length x width2) and mouse weight were recorded every three days and compared between the four groups. Animals were euthanized on the 27th treatment day. Tumors were then harvested, measured, weighed, and photographed. Statistical analysis Data for experiments are expressed as SE or SD of mean (median) values specified for each experiment in corresponding figure legends. Statistical tests were performed using GraphPad Prism (version 7.03; GraphPad Software Inc., San Diego, CA). Tumor volume change was analyzed in Prism using one-way ANOVA. All of the mouse experiments were approved by the Animal Care and Use Committee at Peking University First Hospital.

### Flow cytometry

For cell cycle analysis, cells were seeded and treated with DMSO, Aebmaciclib (1000nM), JQ1(500nM), Abemaiclib (1000nM) + JQ1(500 nm). After 48 h, we fixed the cells in ice-cold 75% ethanol and stored them at -20℃ overnight. The cell pellet was mixed with 3 mL of precooled 70% ethanol, and fixed at -20℃ overnight. The next day, we centrifuge at 1000 g for 5 min to precipitate the cells, and then carefully suck out the supernatant. Add 1 mL of precooled PBS and resuspend twice. We then rewashed the cells and added 0.5 ml propidium iodide staining solution (propodium iodide 20ug/ml + RNAse A 100ug/ml) to each tube cell sample, gently mixed and resuspend the cell pellet, and incubated at 37℃ for 30 min in the dark before analyzing them on a FACSCalibur (BD Biosciences).

### SA-β-gal staining

Senescence β-Galactosidase (SA- β-Gal) activity was evaluated with a β-galactosidase staining kit from Beyotime (#C0602). For the tissue staining, equilibrate the frozen tissue to room temperature. Rinse the tissue with PBS 3 times for 5 min each time. Add an appropriate amount of β-galactosidase Staining Fixative to fully cover the tissue and incubate at room temperature for 15 min. And then rinse the tissue with PBS 3 times for 5 min each time. Remove the PBS, add an appropriate amount of staining working solution. Incubate overnight at 37℃, and the nest day, examine by a light microscope.

### Quantitative real-time PCR

RNA was extracted from mouse tumor samples using TRIzol (Invitrogen) RNA extraction protocol and reverse-transcribed to cDNA using a RT-PCR kit (TIANGEN, #KR116). Real-time quantitative PCR was performed using SYBR Green PCR reagent (TIANGEN, #FP205) using the following PCR cycle parameters: 95 °C for 15 min followed by 40 cycles of 95 °C for 10 s, 55 °C for 30 s, and 72 °C for 30 s with final extension at 72 °C for 10 min. GAPDH was used as control for comparison. Primers are listed in supplementary Table S3.

### RNA-seq and analysis

Total RNA was isolated from cell lines following TRIzol (Invitrogen) RNA extraction protocol. Library preparation and RNA-seq were performed by Novogene using their paired-end sequencing pipeline. Paired-end reads were generated on Illumina Hiseq 2500 platform. After quality control, clean data were aligned to UCSC hg19 reference by STAR and quantified using RSEM with default paraments. Differentially expressed genes were determined with the R package DESeq2 (RRID:SCR_000154). Pathway enrichment analysis of GO, KEGG, REACTOME, and HALLMARK database, as well as gene set enrichment analysis (GSEA) were performed using ClusterProfiler (RRID:SCR_016884) package.

### CUT&Tag Seq and analysis

CUT&Tag assay was performed on AGS cells treated with DMSO and ABE for 24 h following the manufacturer’s protocol using the Hyperactive Universal CUT&Tag Assay Kit for Illumina (Vazyme). Raw data were aligned to hg19 using bowtie2 (RRID:SCR_016368) and peaks were called by SEACR. Super enhancers were determined by ROSE and enrichment analysis was performed with ChIP-Enrich. Visualization of CUT&Tag data was generated using deepTools (RRID:SCR_016366) and ggplot2.

### Public datasets

Genomic and transcriptomic datasets of TCGA-STAD and 36 gastric cancer cell lines were downloaded from cBioPortal. IHC staining results of CDK4, CDK6, and BRD4 were obtained from the Human Protein Atlas. Pan-cancer genomic dependency data was obtained from the DepMap portal.

### Statistical analysis

For the comparison of expression level in TCGA-STAD cohort, Student’s t test was used. Differentially expression genes were determined by DESeq2 and adjusted *p* value < 0.05 was used as one of the thresholds. Pathway enrichment analysis (statistical testing based on hypergeometric distribution) and Gene Set Enrichment Analysis (statistical testing based on permuted empirical distribution) embedded in the ClusterProfiler R package were used for enrichment analysis. Wilcox test was used for comparison between drug-treated assays which were conducted in three replicates. DNA damage repair pathway network was built in STRING (web server) and further optimized by Cytoscape.

## Results

### Pharmacological inhibition of CDK4/6 in gastric cancer cell lines

**Fig. 1:**
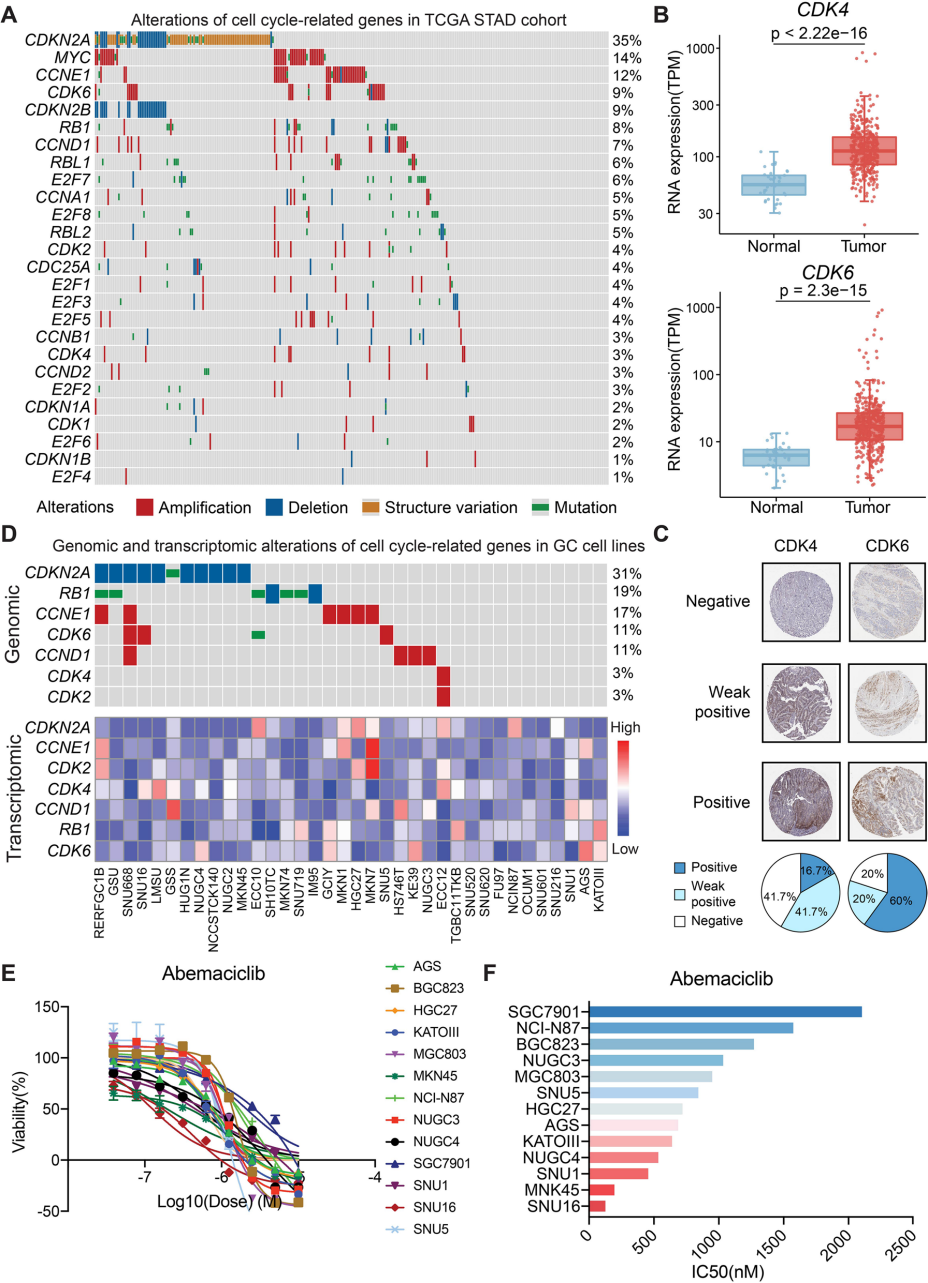
Alterations and pharmacological inhibition of CDK4/6 in GC cell lines. **A** Percentage aberrations in cell cycle-related genes shown in the TCGA-STAD cohort (PanCancer Atlas). **B** Gene Expression Profiling Analysis data from the TCGA databases of TCGA-STAD dataset demonstrated elevated mRNA levels of CDK4 and CDK6 in GC compared with adjacent normal tissue. **C** Immunohistochemistry (IHC) staining of CDK4 and CDK6 in GC patients from The Human Protein Atlas. **D** Percentage aberrations and mRNA expression in cell cycle-related genes shown in the GC cell lines. **E** Dose-response curves for ABE in 13 GC cell lines. ABE was evaluated in 10 serially diluted doses and each dose was analyzed in technical quadruplicate in each biological replication. The relative cell number was determined using the CellTiter-GLo signal captured on the luminescent microplate reader. **F** Average IC50 values generated from dose-response curves for ABE

Alterations in CDKN2A-CDK4/6/CCND1 machinery were frequently observed in gastric cancer patients (TCGA) (Fig. 1A) [9]. Notably, CDK4 and CDK6 were significantly upregulated in gastric tumors than adjacent normal tissues (Fig. 1B) and were dysregulated among GC patients (Fig. 1C) [30]. These genomic and transcriptomic alterations were clearly recapitulated by various GC cell lines [31], making them ideal preclinical models for exploring pharmacological effects of CDK4/6 inhibitors and drug screening for combination therapies in GC (Fig. 1D).

To evaluate the sensitivity of GC toward CDK4/6 inhibition, we performed the drug-dose response assays among a panel of 13 human GC cell lines treated with abemaciclib (abbreviated to ABE hereafter) (Fig. 1E). Treatment with ABE showed antigrowth activity in all 13 gastric cancer cell lines with a median IC50 of 856.1 ± 152.2 (range from 126.2 to 2104 nM) (Fig. 1E and F). These results suggested that ABE showed pharmacological inhibition in the majority of human GC cell lines and was capable of further evaluation in combinations.

### Drug screening proposes a synergistic combination of ABE and JQ1

**Fig. 2:**
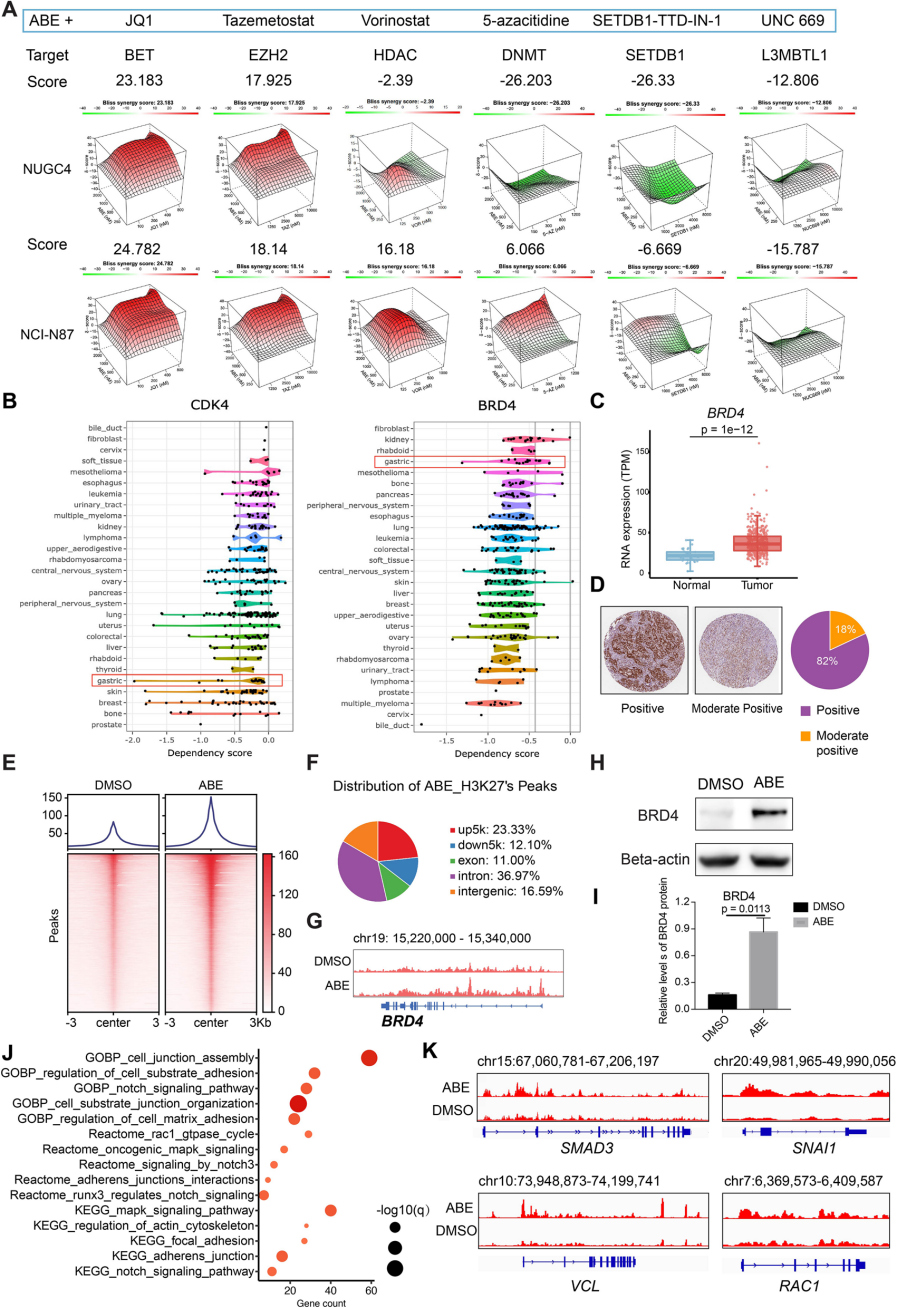
JQ1 is a promising candidate in combination with ABE. **A** Ranked synergy models of ABE and screened epigenetic drugs, including JQ1, tazemetostat, vorinostat, 5-azacitidine, SETDB1-TTD-IN-1 and UNC669. Synergy scores were labelled to the top of each model. **B** Dependency score of CDK4 and BRD4 for tumor cells across different cancer types. Score < 0 represents essential genes and Score < -1 represents super essential genes. **C** Transcriptional expression level of BRD4 between tumor and normal tissues in TCGA-STAD cohort. **D** Immunohistochemistry (IHC) staining of BRD4 in GC patients from The Human Protein Atlas. **E** CUT&Tag-seq binding enrichment of H3K27ac in AGS cells treated with DMSO or ABE. **F** Pie plot of the genomic distribution of H3K27ac peaks in ABE-treated AGS cells. **G** Gene tracks depicting H3K27ac signals at the BRD4 locus. The signal from ABE and DMSO was normalized at same level. **H** Western blotting images showing the protein level of BRD4 protein level after ABE treatment. GAPDH was used as a loading control. **I** Quantification of the protein level of BRD4 in AGS cells after ABE treatment for 24 h using ImageJ software. The data are presented as the mean ± SEM of three replicates. **J** Enrichment analysis of top1000 upregulated H3K27ac peaks in ABE-treated AGS cells. **K** Gene tracks depicting H3K27ac signals at the SMAD3, VCL, SNAI1 and RAC1 locus. The signals from ABE and DMSO were normalized at same level

Previous studies and clinical trials demonstrated that CDK4/6 inhibitors are more likely to be treated in a combination therapy rather than monotherapy [19, 32–35]. Notably, CDK4/6 inhibitors are reported to exhibit good antitumor effects in combination with epigenetic regulatory drugs [23]. Therefore, we screened the combination effects between ABE and 6 drugs targeting various regulators of epigenetic process, including JQ1 (target: BRD4), TAZ (EZH2), Vorinostat (HDAC), 5-azacitidine (DNMT), SETDB1-TTD-IN-1 (SETDB1), and UNC669 (L3MBTL1) (Fig. 2A). We performed a drug screening to identify targeted epigenetic inhibitors that potentiate response to CDK4/6 inhibitor in two GC cell lines to identify improved treatment strategies. The cells were treated 3 days against a 5-point dose range of the epigenetic compound combined with a 5-point dose concentration of CDK4/6 inhibitor and then tested for cell viability using the CellTiter-Glo Luminescent reagent. (Fig. 2A and Materials and methods). We used Bliss model with SynergyFinder [36] to calculate synergy score and reveal combinations that specifically induced higher cell death than single agents. Notably, the BRD4 inhibitor in the screen (JQ1) achieved the best synthetic lethal effect of ABE in two GC cell lines (synergy score, 24.67 in NUGC4 and 23.17 in NCI-N87), suggesting BRD4 as a candidate combination therapeutic target for ABE (Supplementary Fig. S1A).

Pan-cancer analysis revealed that both CDK4 and BRD4 are super essential genes (Gene Effect score < -1) for gastric cancer cells (Fig. 2B) [37]. BRD4 was screened out in more than 50% of gastric cancer cell lines (19/35). Furthermore, 14 out of 19 cell lines enriched for BRD4 are also enriched for genes related to the cell-cycle axis, which shows that BRD4 inhibition and cell cycle inhibitor are more likely to be associated with joint lethality (Supplementary Fig. S1B). BRD4, a well-established reader for H3K27ac [38] reported to promote gastric cancer progression and metastasis [39], was elevated in gastric tumors (Fig. 2C and D) and was correlated to inferior prognoses of GC patients [39]. To further explore the regulation relationship between CDK4 and BRD4, we performed Cleavage Under Target & Tagmentation sequencing (CUT&Tag-seq) for H3K27ac, a mark of active enhancers, in the presence and absence of ABE. As a result, more H3K27ac peaks were obtained in GC cells treated with ABE (Fig. 2E). Notably, the level of H3K27ac was elevated at BRD4 loci after CDK4/6 inhibition, implying an activation of BRD4 in response to ABE (Fig. 2G). Consistently, the protein level of BRD4 was significantly increased after ABE treatment (Fig. 2H and I). Further, over half of peaks in ABE-treated cells were located within distal intergenic regions or introns, suggesting that they might represent activated enhancers (Fig. 2F) [40]. Given the vital role of BRD4 in super-enhancer (SE) organization [41–43], we next want to define the expression-activated gene signature in ABE-treated GC cells. The enrichment analysis of these peaks in GO, KEGG and Reactome uncovered that cell junction and cell matrix adhesion regulation were significantly upregulated after ABE treatment (Fig. 2J). Notably, the level of H3K27ac was elevated at cell junction related gene loci including *SMAD3, VCL, RAC1* and *SNAI1* after CDK4/6 inhibition (Fig. 2K). Altogether, these results suggested that CDK4/6 inhibitors induce chromatin remodeling in gastric cancer cells characterized by the extensive enhancer activation and proposed that BRD4 was a promising combination target for ABE.

### BRD4 inhibition sensitizes gastric cancer to CDK4/6 inhibition

**Fig. 3:**
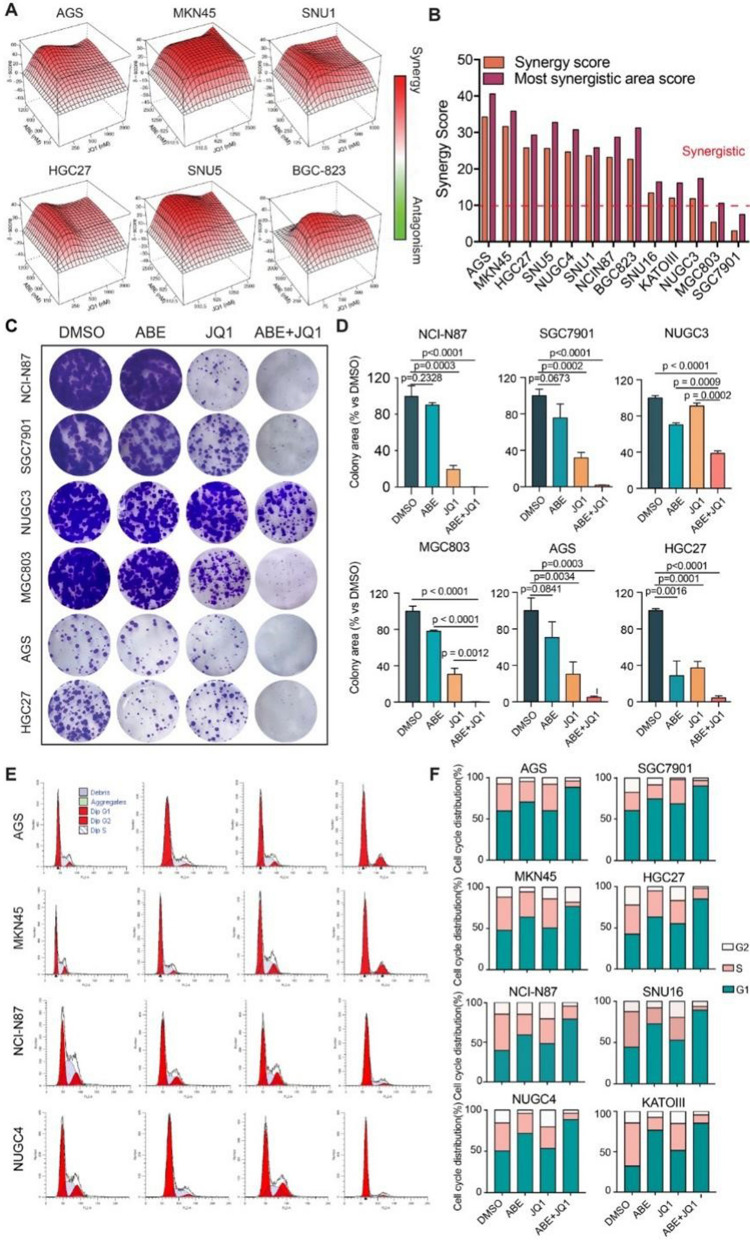
Cellular effects of the combination of ABE and JQ1 on in vitro GC cell models. **A** Representative synergy models of ABE and JQ1 across GC cell lines. **B** Bar plot of the average and maximum of synergy score among 13 GC cell lines. score > 10 indicates synergy. score < -10 indicates antagonism. **C** Crystal violet staining of colonies from six representative cell lines during 2 weeks with the indicated treatment of DMSO, ABE (100 nM), JQ1(100 nM), and ABE (100 nM) + JQ1 (100 nM). **D** Quantification of the colonies area using imageJ software. The data are presented as the mean ± SEM of three replicates. **E** Representative Cell cycle plots of different GC cell lines treated with DMSO, ABE (1000 nM), JQ1(500 nM), or ABE (1000 nM) + JQ1(500 nM) for 48 h as examined by flow cytometry analyses. **F** Representative histograms of the ratio of G1, S, and G2 phase of GC cell lines with different treatments

To explore the synergism between ABE and JQ1 across various GC cell lines, we first determined the IC50 of JQ1 on 13 GC cell lines (Supplementary Fig. S2A and B, Tables S1 and 2), including AGS, HGC27, BGC-803, KATO III, MKN45, SNU1, SNU16, NUGC3, MGC803, SNU5, NUGC4, NCI-N87 and SGC7901 (Fig. 3A; Supplementary Fig. S2C). These cell lines mimicked the genomic characteristics of heterogeneous gastric tumors. Strikingly, combenefit analysis obtained an average synergy score of 19.78 ± 2.693 (highest in AGS:34.29) for the combination of ABE and JQ1 (Fig. 3B), indicating strong synergistic cooperations of ABE and JQ1 in all tested cell lines.

To further elucidate the pharmacological potency of the combination of ABE and JQ1, we performed the colony formation assay (Fig. 3C). Though GC cell lines responded differently to either monotherapy, a consistent sensitivity gain was observed in all cell lines treated with the combination of ABE and JQ1 (Fig. 3D). This result provided an important clinical clue that the combination of ABE and JQ1 was necessary to overcome possible poor efficacy for either monotherapy and demonstrated superior antitumor effects.

We also performed flow cytometric analysis of cell cycle in GC cells treated with DMSO, ABE, JQ1, and ABE + JQ1 (Fig. 3E; Supplementary Fig. S2D). We noted that ABE treatment of GC cell lines resulted in an increase in the percentage of G1 phase cell population (69.11 ± 6.25) as compared to untreated cells (Fig. 3F), while treatment with JQ1 displayed a modest increase in G1 phase (55.15 ± 6.50). Notably, the combination treatment induced a significant increase in percentage of G1 phase cells (85.34 ± 4.98), suggesting a sensitizing effect of JQ1 on the cell cycle arrest induced by ABE.

Taken together, these results demonstrated that inhibition of *BRD4* via pharmacological agents induced a synergistic effect in combination with the *CDK4/6* inhibitor ABE, resulting in growth inhibition and cell cycle arrest in GC cells.

### BRD4 inhibition specifically enhances the cancer cell killing effects of CDK4/6 inhibitors by inducing cell senescence

**Fig. 4:**
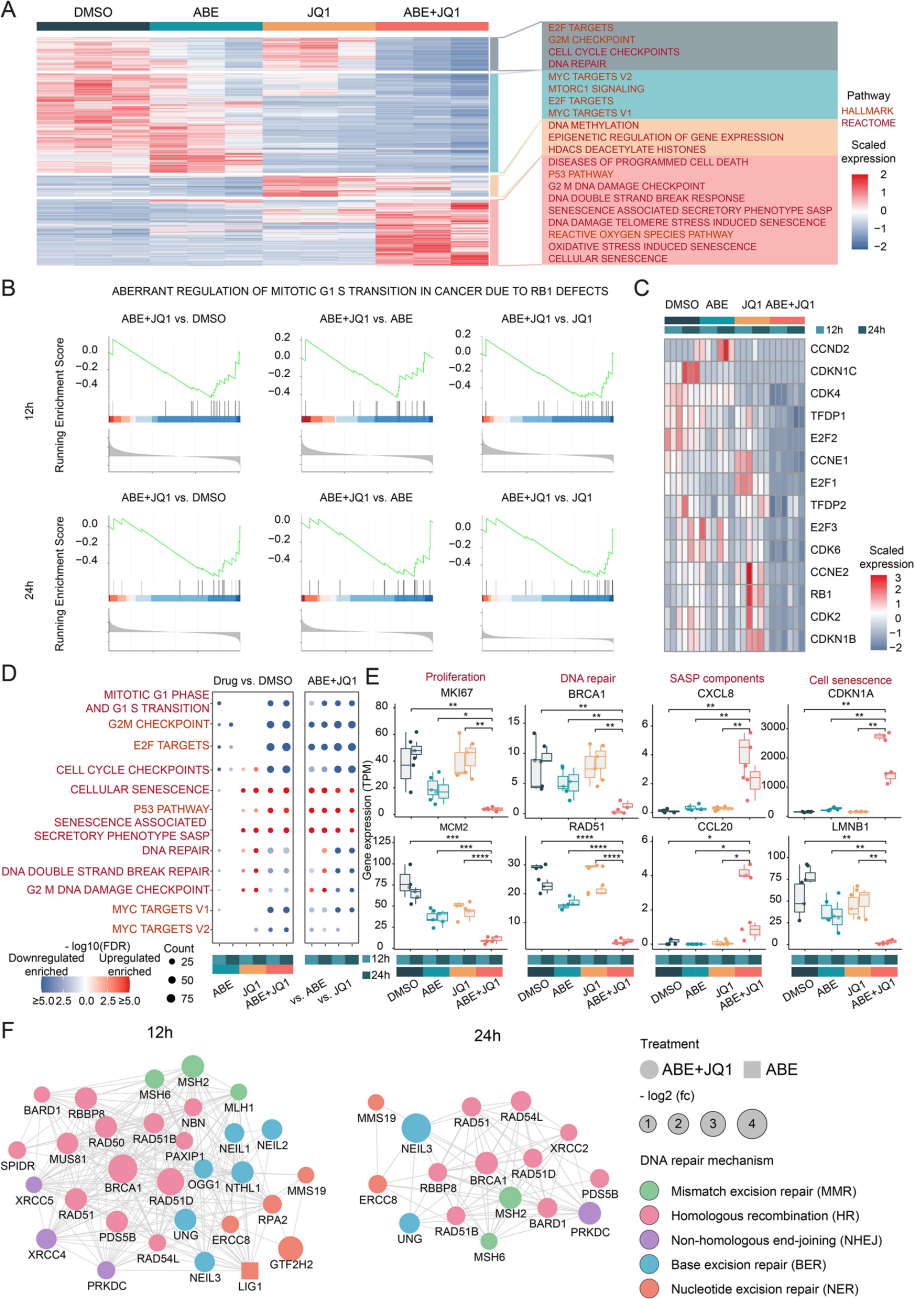
Molecular mechanism of the combination of ABE and JQ1 in GC cell lines revealed by RNA-seq. **A** Heatmap of differential expressed genes among AGS cells with different treatments and time points. HALLMARK and REACTOME functions are annotated on the right. **B** GSEA plots of ABERRANT REGULATION OF MITOTIC G1 S TRANSITION IN CANCER DUE TO RB1 DEFECTS of comparisons among different treatments and timepoints. **C** Heatmap of cell cycle-related genes. **D** Dot plot of enriched pathways in different comparisons. In each comparison, pathways enriched in upregulated genes are colored in red and those enriched in downregulated genes are in blue. **E** Box plots showing the expression levels of marker genes of PROLIFERATION (MKI67, MCM2), DNA REPAIR (RAD51, BRCA1), CELLULAR SENESCENCE (CDKN1A, LMNB1) and SASP (CXCL8, CCL20). **F** Network of genes involved in five DNA repair pathways of the indicted cohort. JQ1 vs. DMSO cohort has no related downregulated DNA repair genes. Colors denote different pathways. The dot size is corresponding to fold change. Different shapes denote different treatments

To interrogate the mechanism of synthetic lethality behind the combination of ABE and JQ1, we portrayed the transcriptomic profiling of AGS cells under different four cohort of treatments (DMSO, ABE, JQ1, ABE + JQ1) at 12 and 24 h (Figs. 4A, S3A and B). Consistent with previous reports, genes involved in cell cycle such as E2F targets and G2M checkpoint were significantly downregulated in ABE-treated cells [44]. Meanwhile, JQ1-treated cells showed a dysfunction of epigenetic regulation [45]. Notably, compared with either monotherapy, the combination of ABE and JQ1 induced a significantly more severe suppression of cell cycle, which was accordant at both time points (Fig. 4B and C, Supplementary Fig. S3G). These results suggest that BRD4 inhibition enhances the cell cycle arrest effect of CDK4/6 inhibitor.

Moreover, combination of ABE and JQ1 induced unique characteristics of cellular senescence (Fig. 4A and D; Supplementary Fig. S3C and H). Cellular senescence is a state of irreversible cell cycle arrest triggered by inducers such as persistent DNA damage, ROS accumulation, oncogene activation and telomere dysfunction [46–48]. Significantly upregulations of CDKN1A and downregulation of LMNB1 --- classical markers for cellular senescence [48, 49], were observed after combinative treatment (Fig. 4E). We further noted a plethora of factors termed the senescent associated secretory phenotype (SASP), such as pro-inflammatory cytokines and chemokines (IL-8, CCL20), growth modulators (TGFB1), proteases and regulators (TIMP1 and SERPINE1), and matrix metalloproteinases (MMP1, MMP3, MMP10), were significantly upregulated after combinative treatment (Fig. 4E; Supplementary Fig. S4B). In addition, gastric tumor cells exhibited evident DNA damage and DNA repair defect, supported by downregulation of well-known genes involved in DNA damage and repair response, including RAD51, BRAC1, BRCA2 and BARD1 (Fig. 4E; Supplementary Figs. S3D-F; S4A) [50, 51]. Detailed investigation of DNA repair mechanism showed that homologous recombination, rather than other DNA repair pathways, was impaired most under the combination of ABE and JQ1 (Fig. 4F, Supplementary Fig. S3F). Taken together, these results indicated that the synthetic lethal of ABE and JQ1 is mediated by cellular senescence stimulated by constant DNA damage and ROS accumulation.

### BRD4 inhibition cooperates with CDK4/6 inhibition to induce cell senescence and DNA damage in vitro

**Fig. 5:**
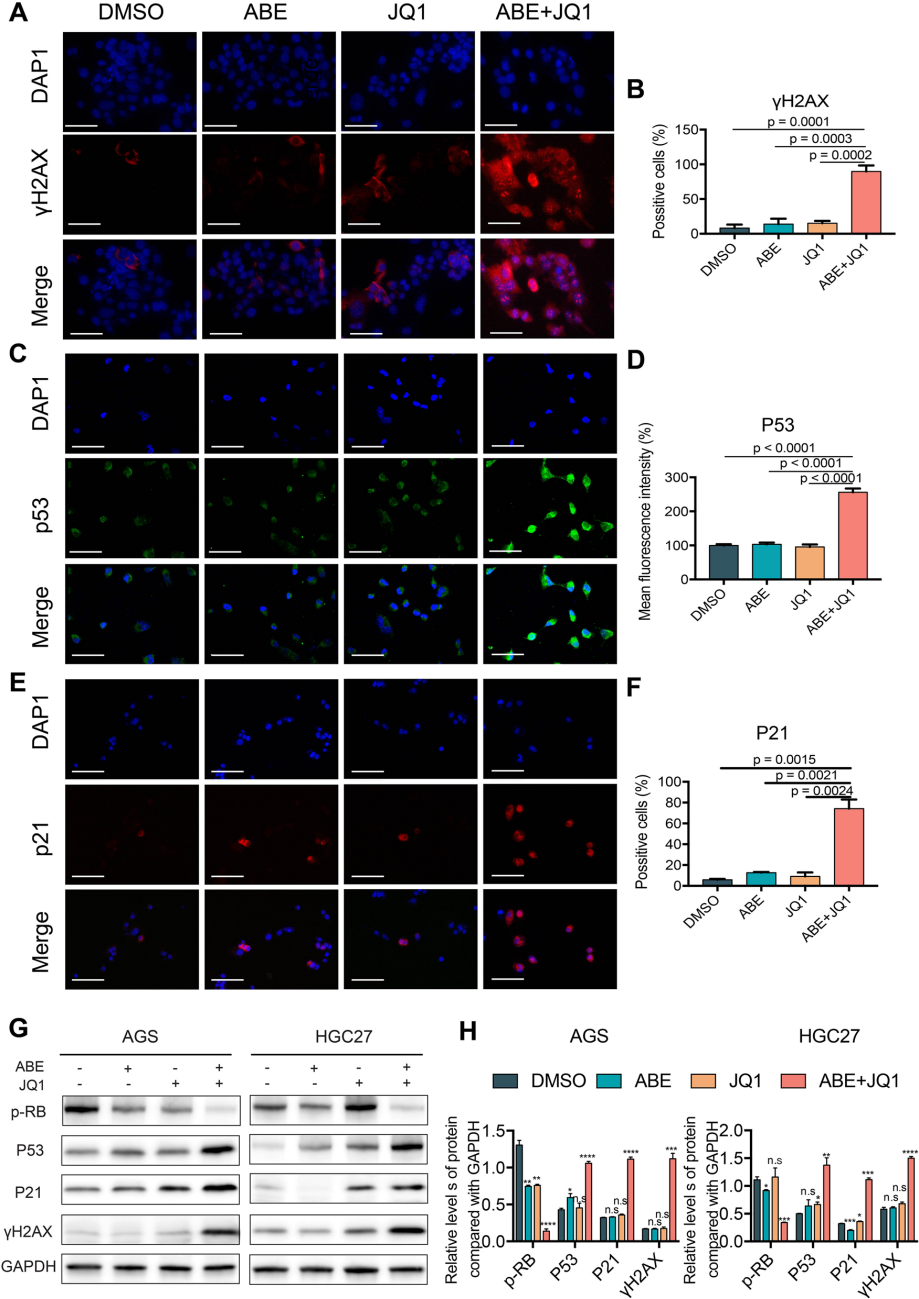
CDK4/6 and BRD4 Inhibition Specifically induce cell senescence and DNA damage in vitro. **A**, **C**, **E** Immunofluorescence staining of the DNA damage marker γH2AX (scale bar = 25 μm), DNA senescence marker p21 and p53 (scale bar = 50 μm ) in AGS cell line upon the treatment of the indicted agents for 24 h. **B**, **D**, **F** Quantification of the positive cell ratio and mean fluorescence intensity using QuPath software. The data are presented as the mean ± SEM of three replicates. **G** Western blotting images showing the protein level of Phospho-Rb (Ser807/811), γH2AX, p53 and p21 in AGS cells from the indicted treatment cohort. GAPDH was used as a loading control. **H** Quantification of the protein level of Phospho-Rb (Ser807/811), γH2AX, p53 and p21 in AGS cells from the indicted treatment for 24 h using imageJ software. The data are presented as the mean ± SEM of three replicates. Significance of each group was compared with DMSO group

To validate the DNA damage and cellular senescence specifically induced by the combined treatment of ABE and JQ1 in GC, we performed immunofluorescence (IF) staining and western blot (WB) analysis with a series of well-established markers. DNA damage level was significantly elevated in combination-treated cells as shown by upregulation of γH2AX foci (Fig. 5A, B, G). A significant increase of P53 fluorescent intensity in doublet-treated cells (256.3% ± 10.78%) was also observed as compared to the ABE-monotherapy (103.1% ± 5.38%) and JQ1-monotherapy (95.36% ± 7.284%) (Fig. 5C and D). Western blot further validated the upregulation of P53 at protein level (Fig. 5G), further supporting the increased DNA damage and cellular senescence in doublet-treated GC cells. In addition, P21, another senescence marker, was also detected to be clearly increased in combination-treated cells by both IF staining and WB analysis (Fig. 5E-G). In addition, downregulation of phosphorylated RB ((Ser807/811) indicates severe cell cycle inhibition by the combination the ABE and JQ1 (Fig. 5G and H). These results confirmed that combination of ABE and JQ1 could induce DNA damage, cellular senescence and cell cycle arrest in GC cells as we observed at RNA level.

### Combination of ABE and JQ1 shows potent antitumor effects in vivo

**Fig. 6:**
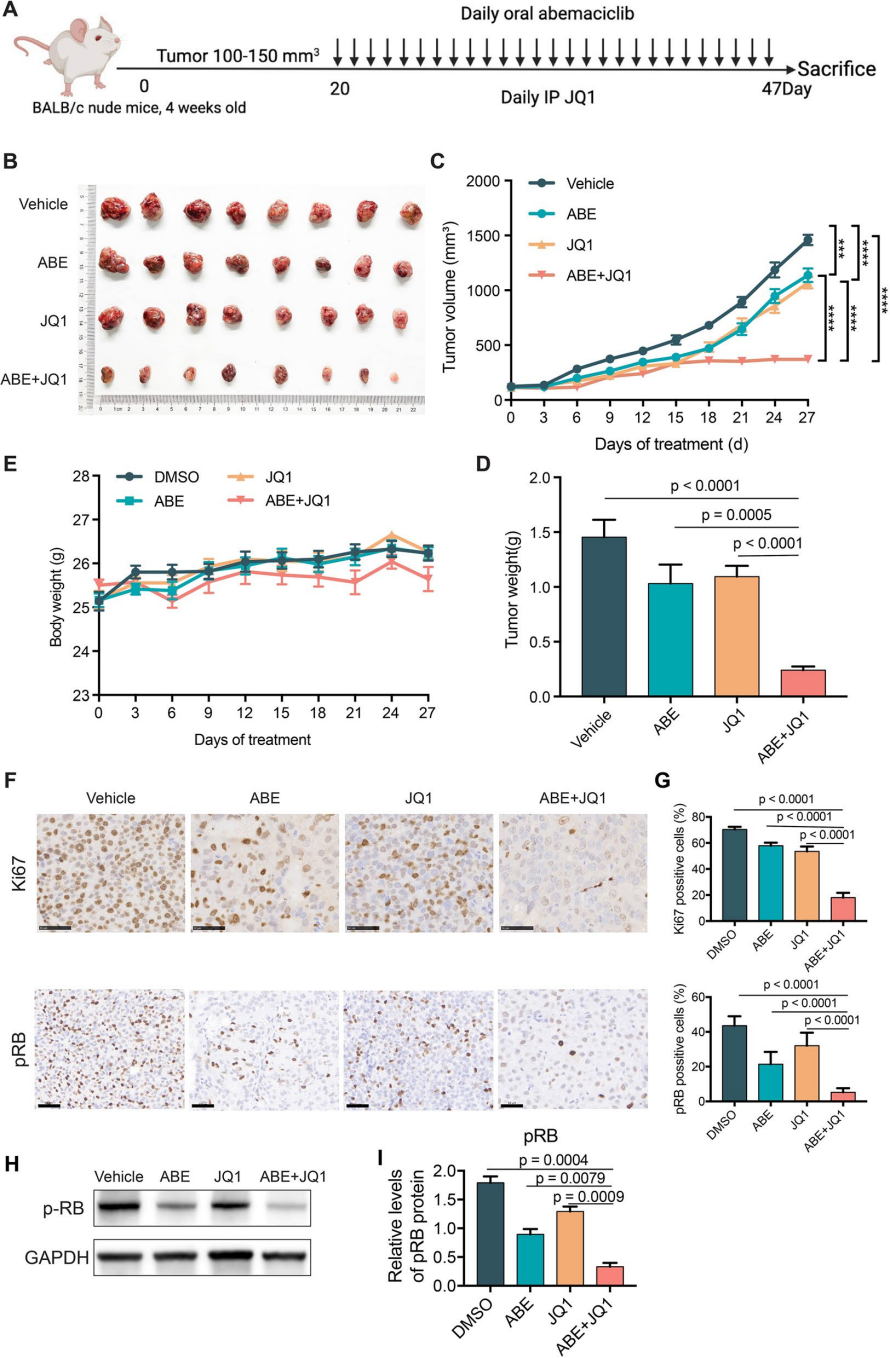
ABE and JQ1 were synergetic in vivo through inducing cell cycle arrest and cellular senescence. **A** Schemes for the in vivo experimental procedures to evaluate anticancer activities of ABE and JQ1. **B**, **C** Combination of ABE and JQ1 treatment improved the antitumor efficacy of Abemaciclib agents in vivo in the HGC27 tumor models. HGC27 cells (5 × 106/mouse) were subcutaneously injected into Balb/c- nu mice. Tumor-bearing mice received solvent control, abemaciclib (50 mg kg − 1 body weight) by oral, JQ1 (35 mg kg − 1 body weight) by intraperitoneal injection. Tumor volumes shown in (**C**) were measured and presented as mean ± SEM (*n* = 8 mice/group). Representative images of the xenograft tumors obtained from the indicated groups at the endpoint of the experiments (day 27) are shown in (**B**). **D** Histogram results showing the mean ± SEM of the tumor weights from the indicated groups at the endpoint of the experiments. **E** Line chart showing the mouse weight from the indicated groups. **F** Representative images for indicated assays in the end of study tumors showing the immunohistological evaluation of cellular proliferation by Ki-67 staining, the bottom panel shows the RB phosphorylation, each for the treatment cohort as labeled. Scale bar = 50 μm. **G** Statistics data represent mean ± SEM (*n* = 10) of Ki67 and pRB for each group. **H** Western blotting images showing the pRB in tumor lysates derived from the indicted treatment cohort. GAPDH was used as a loading control. **I** Statistics data represent mean ± SEM (*n* = 3) of Phospho-Rb (Ser807/811) protein level for each group

To determine the in vivo efficacy of the combination of ABE and JQ1, we deployed the CDX model using the GC cell line HGC27 (Fig. 6A). Compared to either monotherapy, the combination treatment of ABE and JQ1 significantly restricted tumor growth in HGC27 xenograft model (Fig. 6B and C). Consistently, the tumor weight was also sharply reduced under the combination treatment of ABE and JQ1 (Fig. 6D). Meanwhile, all mice tolerated the treatment well during the dosing period, as mice in the combined cohort weighed slightly less than the control group, but the difference was not statistically significant (Fig. 6E). These results collectively demonstrated the in vivo antitumor activity of the combination treatment for GC. The immunohistochemical (IHC) analysis of tumors isolated from mice at the treatment endpoints revealed that Ki67 expression was significantly suppressed in combination-treated cohort (Fig. 6F and G). Pathological and WB analysis showed that the tumors in combination group exhibited apparent inhibition of *CDK4/6* activity as the phosphorylation of RB (pRB, Ser807/811) in the combination group was efficiently reduced (Fig. 6H and I), suggesting an evident cell cycle arrest and tumor proliferation restriction.

### Evaluation of pharmacodynamic and functional markers of doublet combination in in vivo GC models

**Fig. 7:**
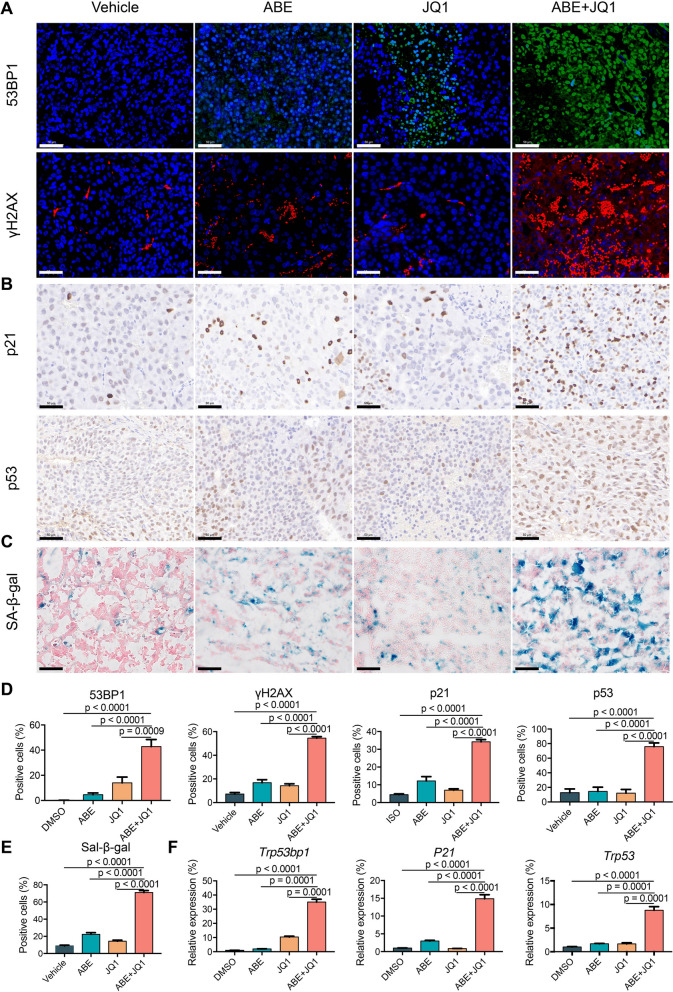
The combination of ABE and JQ1 induced cell senescence and DNA damage in vivo. **A** Immunofluorescence staining of the DNA damage marker 53BP1 and γH2AX in tumor samples derived from the HGC27 subcutaneous mouse model upon the treatment of the indicted agents. Scale bar = 50 μm. **B** Immunohistochemical staining of the DNA senescence markers p21 and p53 in tumor samples derived from the HGC27 subcutaneous mouse model upon the treatment of the indicted agents. Scale bar = 50 μm. **C** Senescence β-Galactosidase Staining in tumor samples derived from the HGC27 subcutaneous mouse model upon the treatment of the indicted agents. Scale bar = 50 μm. **D** Quantification of the positive cell ratio using QuPath software. The data are presented as the mean ± SEM of ten replicates. **E** Quantification of the SA-β-Gal positive cell ratio using QuPath software. The data are presented as the mean ± SEM of ten replicates. **F** The RNA expression level of P21, TP53 and 53BP1 detected by real-time PCR. The data are presented as the mean ± SEM of three replicates

To assess whether the DNA damage and cellular senescence can be recapitulated in vivo, we performed the immunohistochemical and immunofluorescence on the tumor tissues from four groups of treatments (Fig. 7A and B). Our results showed that the combination remarkably induced DNA damage and cell senescence evidenced by the upregulation of P53, P21, γH2AX and 53BP1 (Fig. 7D). These results were further supported by the strongest staining of the classical cell senescence marker, senescence-associated beta-galactosidase (SA-β-Gal), in the GC mice treated with the combination of ABE and JQ1 (Fig. 7C and E). In addition, Real-time PCR assays corroborated the overexpression of these genes at transcriptomic level (Fig. 7F).

## Discussion

Although progress is being made, treatment options for patients with advanced GC are still limited [4, 6, 52]. By far only two targeted therapeutic agents, trastuzumab and ramucirumab, have been approved by the Food and Drug Administration with modest Overall Response Rate (ORR) improvement [5]. Previous studies have revealed evident alterations of cell cycle genes, especially cyclin-dependent kinases, in over half of GC patients among various cohorts, implying a promising therapeutic candidate for those GC patients resistant to first-line therapies [53–56]. Several CDK4/6 inhibitors, the most clear-cut success of drugs targeting cell cycle, have entered phase 1 and phase 2 clinical trials for the treatment of GC as monotherapy or in combination with other therapies (NCT02378389, NCT03480256, NCT03891784) [57]. Consistent with previous reports in other cancer types, treatment with a single CDK4/6 inhibitor was ineffective in many GC patients [58]. In contrast, CDK4/6 inhibitors have been found to be more effective in combination with other drugs that may potentially circumvent the limitation [15, 59–63]. Thus, candidate targets were required for in combination with CDK4/6 inhibitors to enhance the effectiveness of targeted therapies in GC [64].

Epigenetic aberration is an important hallmark of gastric cancer, which was observed in both onset and development phase of GC [65, 66]. Several epigenetic strategies have been proposed for the treatment of GC including DNMTs (5-azacitidine), HDACs (Vorinostat) and HMTs (EZH2) in preclinical studies [67–71]. Here, we performed a comprehensive epigenetic drug screening, CUT&Tag sequencing, and Depmap CRISPR data profiling analyses to identify synthetic lethal targets with ABE in GC. Strikingly, the BRD4 inhibitor JQ1 outperformed a dozen of epigenetic drugs with the highest synergy score of 24.67 in NUGC4 and 23.17 in NCI-N87. BRD4, well-known for its role in super-enhancer organization and transcriptional activation, was elevated in gastric tumors and was correlated to inferior prognoses of GC patients. In addition, our result showed that inhibition of CDK4/6 induced a significant increase of H3K27ac peaks and hyperactivation of BRD4. Altogether, these results provide a rationale for the combination treatment of CDK4/6 inhibitor ABE and BRD4 inhibitor JQ1 for GC patients.

Of note, we observed evident synergistic effects for the combination of ABE and JQ1 in both in vitro and in vivo GC models. Experiments performed on 13 GC cell lines showed that the capability of GC cells were significantly restricted and the proportion of GC cells arrested in cell cycle G1 were remarkably elevated under the combination treatment of ABE and JQ1. In vivo studies further validated the superior antitumor effect than either monotherapy, and no evident adverse drug reactions were observed in doublet-treated mice. Our results provide important preclinical supports for combination therapies with CDK4/6 inhibitors for patients with gastric cancer.

Finally, our data revealed the novel mechanisms behind the synergism of the doublet combination. On the one hand, JQ1 enhanced the cell cycle arrest induced by ABE in GC cells, demonstrated by the elevated proportion of G1 cells. On the other hand, unique pattern of dysregulation of DNA damage and cellular senescence was observed in the doublet combination compared to either monotherapy, which has been less reported in other cancer types. Transcriptomic analysis showed that the addition of JQ1 to ABE resulted in a significant upregulation of a plethora of factors thought to be secreted by senescent cells, including pro-inflammatory cytokines and chemokines, growth modulators, angiogenic factors, and matrix metalloproteinases (MMPs), suggesting a senescent associated secretory phenotype (SASP) in these GC cells. Additionally, DNA damage and impaired DNA damage repair were also detected under the combined treatment of ABE and JQ1. These results were further validated at protein level by IHC and WB in both in vitro and in vivo models. Collectively, these results suggest that the BRD4 inhibitor JQ1 augments the antitumor efficacy of ABE in preclinical models of gastric carcinoma via inducing cellular senescence.

In summary, BRD4 inhibitor JQ1 was screened out for use in combination with ABE for synthetically lethal to GC cells. Drug combination with ABE and JQ1 showed promising synergistic effects in various in vitro and in vivo GC models. Significant tumor regression was observed in the doublet therapy of ABE and JQ1. Further investigation revealed that JQ1 augmented cell cycle arrest of GC cells resulted from CDK4/6 inhibition and the doublet combination induced obvious cellular senescence. Our results provided sufficient experimental evidence and detailed mechanistic investigation to support a novel combination therapy strategy for the treatment of gastric cancer.

## Conclusion

CDK4/6 inhibitor therapy is a promising targeted treatment for tumor and is being investigated in gastric cancer and a variety of other malignancies. CDK4/6 altered frequently in gastric cancer and CDK4/6 inhibitor ABE showed pharmacological efficacy in numerous preclinical models of gastric cancer. Here, we screened the BRD4 inhibitor JQ1 as the most effective combination candidate for ABE from various epigenetic drugs based on drug screening, whole genome-scale CRISPR-Cas9 dependency analysis, and CUT&Tag sequencing. We validated synergy between ABE and JQ1 utilizing in vitro cell lines and in vivo cell-derived xenograft gastric cancer models. Our results provided sufficient experimental evidence and detailed mechanistic investigation to support a novel combination therapy strategy for the further clinical trials testing for gastric cancer.

### Supplementary Information

**Additional file 1: Figure S1.** Pharmaceutical drug screening and Depmap sgRNA screening indicated that epigenetic inhibitor JQ1 and cell cycle inhibition are more likely to be associated with joint lethality. A) Bar plot of the average and maximum synergy score of abemaciclib combination with 6 epigenetic drugs in 2 GC cell lines. B) Rank plot showing the essential gene of cell-cycle related genes including CDK4, CDK6, CDK2, CCND1 and epigenetic regulator BRD4 in 35 gastric cancer cell lines form depmap dataset. **Figure S2.** Synergistic analysis of ABE and JQ1 for the in vitro tests. A) Dose-response curves for JQ1 in 13 GC cell lines. JQ1 was evaluated in 10 serially diluted doses and each dose was analyzed in technical quadruplicate in each biological replication. The relative cell number was determined using the CellTiter-GLo signal captured on the luminescent microplate reader. B) Average IC50 values generated from dose-response curves for JQ1. C) Representative synergy models of ABE and JQ1 across the indicted GC cell lines. D) Cell cycle of different GC cell lines treated with DMSO, ABE, JQ1, or ABE+JQ1 for 48h examined by flow cytometry analyses. **Figure S3.** The transcriptome profiling analysis of the four cohort treatments of AGS cells. A) Volcano plot showing the up and down regulated genes in the treatment of indicted agents in 12h and 24h, respectively. B) Venn diagram showing differentially expressed genes（DEGs）(q value < 0.05) by abemaciclib, JQ1, or combination compared with control DMSO in 12h and 24h respectively. C -D) The up and down REACTOME enrichment pathways in tumors upon the treatment of abemaciclib combination of JQ1 versus control DMSO treatment (24h). E) Expression levels of cell senescence, cell cycle checkpoint and DNA repair pathway signature genes for the 12h and 24 h treatment of abemaciclib, JQ1 and the combination of abemaciclib and JQ1 in AGS cell line. F-H) GSEA revealed that Homologous Recombination, E2F targets and Cell Senescence gene sets are enriched in ABE+JQ1-treated AGS cells using RNA sequencing data. **Figure S4.** The transcriptome profiling analysis demonstrated the downregulation of proliferation and DNA repair genes and the upregulation of SASP genes in the ABE+JQ1 combination cohort. A) Box plots showing the expression levels of marker genes of PROLIFERATION (*PCNA, MCM7*) and DNA REPAIR (*BARD1*, *BRCA2*). B) Box plots showing the expression levels of marker genes of SASP (*MMP1, MMP3, MMP10, TGFB1, TIMP1, SERPINE1).* **Table S1.** Panel of 13 gastric cancer cell lines used in the study. **Table S2.** In vitro drug dose response for monotherapy abemaciclib and JQ1 in Gastric cell lines. **Table S3.** Real-time pcr primers of p21, p53 and 53bp1 used in this study.

## Data Availability

The data that support the findings of this study are available in Pan-Cancer studies at https://www.cbioportal.org/. The raw sequence data reported in this paper have been deposited in the Genome Sequence Archive in National Genomics Data Center (Beijing, China) under the BioProject PRJCA013192.

## Abbreviations

5-FU: 5-fluorouracil
ABE: abemaciclib
BET: bromodomain and extraterminal
BRD4: Bromodomain Containing 4
Cas9: CRISPR-associated protein 9
CCND1: Cyclin D1
CDK4: cyclin-dependent kinase 4
CDK6: cyclin-dependent kinase 6
CDKN1A: Cyclin dependent kinase inhibitor 1 A
CDKN2A: Cyclin dependent kinase inhibitor 2 A
CDX: Cell Line-Derived Xenograft
CRISPR: Clustered regularly interspaced palindromic repeats
CUT&Tag: Cleavage Under Targets and Tagmentation
DMSO: dimethyl sulfoxide
DNMT: DNA methyltransferase
EZH2: Enhancer of zeste homolog 2
FDA: Food and Drug Administration
FLOT: 5-FU + leucovorin + oxaliplatin + docetaxel
FOLFOX: leucovorin + 5-FU + oxaliplatin
GC: gastric cancer
HDAC: Histone deacetylase
HDACi: histone deacetylase inhibitor
HER2: human epidermal growth factor receptor 2
HMT: histone methyltransferase
IHC: immunohistochemical
L3MBTL1: Lethal(3)malignant brain tumor-like protein 1
MMP: matrix metalloproteinase
ORR: Overall Response Rate
PAL: palbociclib
RIB: ribociclib
SASP: senescent associated secretory phenotype
SE: super-enhancer
SETDB1: SET Domain Bifurcated Histone Lysine Methyltransferase 1
SOX: S-1 + oxaliplatin
TCGA: The Cancer Genome Atlas
VEGF: Vascular endothelial growth factor
WB: western blot
XELOX: oxaliolatin + capecitabine
