# Correction: The BRD4 inhibitor JQ1 augments the antitumor efficacy of abemaciclib in preclinical models of gastric carcinoma

**DOI:** 10.1186/s13046-024-03126-4

**Published:** 2024-07-20

**Authors:** Mei Feng, Hao Xu, Wenyuan Zhou, Yisheng Pan

**Affiliations:** 1 Division of General Surgery, Peking University First Hospital, Peking University, No. 8, Xi Shiku Street, Beijing, 100034 China; 2 Department of Nuclear Medicine, 2NMPA Key Laboratory for Research and Evaluation of Radiopharmaceuticals (National Medical Products Administration), Peking University Cancer Hospital & Institute, Beijing, 100142 China

**Correction: J Exp Clin Cancer Res 42**:**44 (2023)**

10.1186/s13046-023-02615-2

Following publication of the original article [1], the authors identified minor errors in image-typesetting in Fig. 3; specifically in Fig. 3C and D. The image for the NCI-N87 cell line in Fig. 3C (row 1) was mistakenly included. Consequently, the corresponding statistical analysis for NCI-N87 in Fig. 3D also needs to be corrected.

The corrected figure is given below. The correction does not have any effect on the results or conclusions of the paper.

The original article [1] has been corrected.

Incorrect Figure 3.

**Fig. 3:**
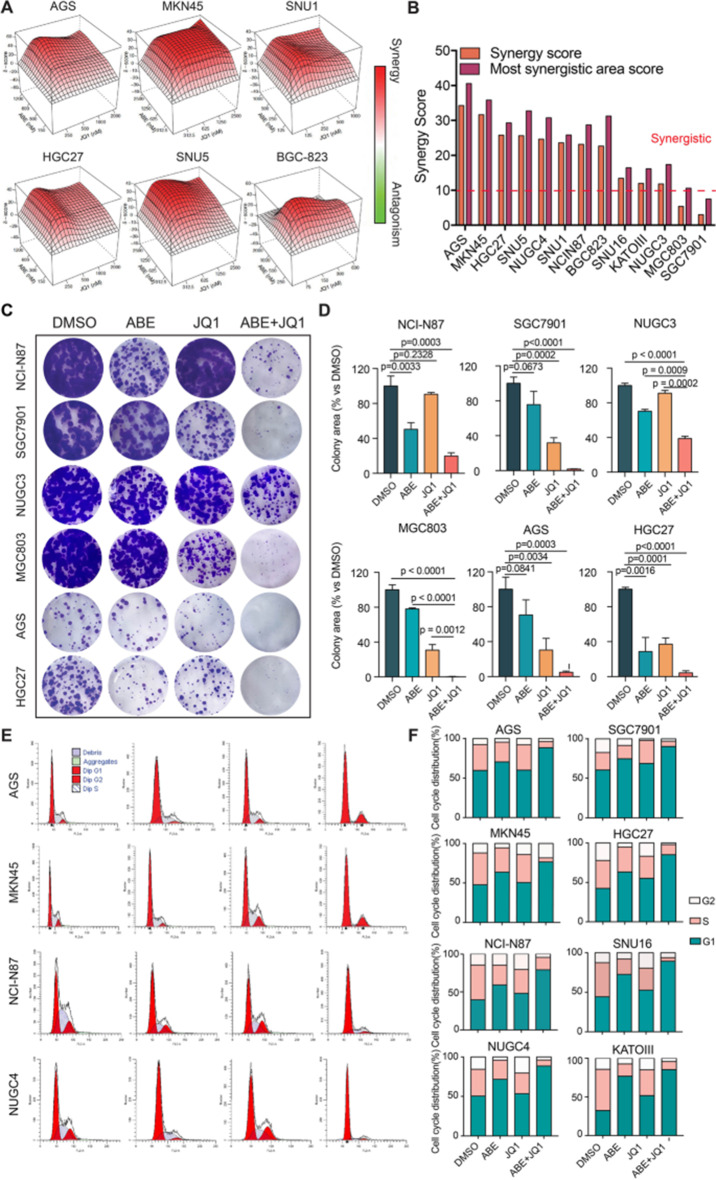
Cellular effects of the combination of ABE and JQ1 on in vitro GC cell models. **A** Representative synergy models of ABE and JQ1 across GC cell lines. **B** Bar plot of the average and maximum of synergy score among 13 GC cell lines. score > 10 indicates synergy. score < -10 indicates antagonism. **C** Crystal violet staining of colonies from six representative cell lines during 2 weeks with the indicated treatment of DMSO, ABE (100nM), JQ1(100nM), and ABE (100nM) + JQ1 (nM). **D** Quantification of the colonies area using imageJ software. The data are presented as the mean ± SEM of three replicates. **E** Representative Cell cycle plots of different GC cell lines treated with DMSO, ABE (1000nM), JQ1(500nM), or ABE (1000nM) + JQ1(500nM) for 48 h as examined by flow cytometry analyses. **F** Representative histograms of the ratio of G1, S, and G2 phase of GC cell lines with different treatments

Correct Figure 3.

**Fig. 3:**
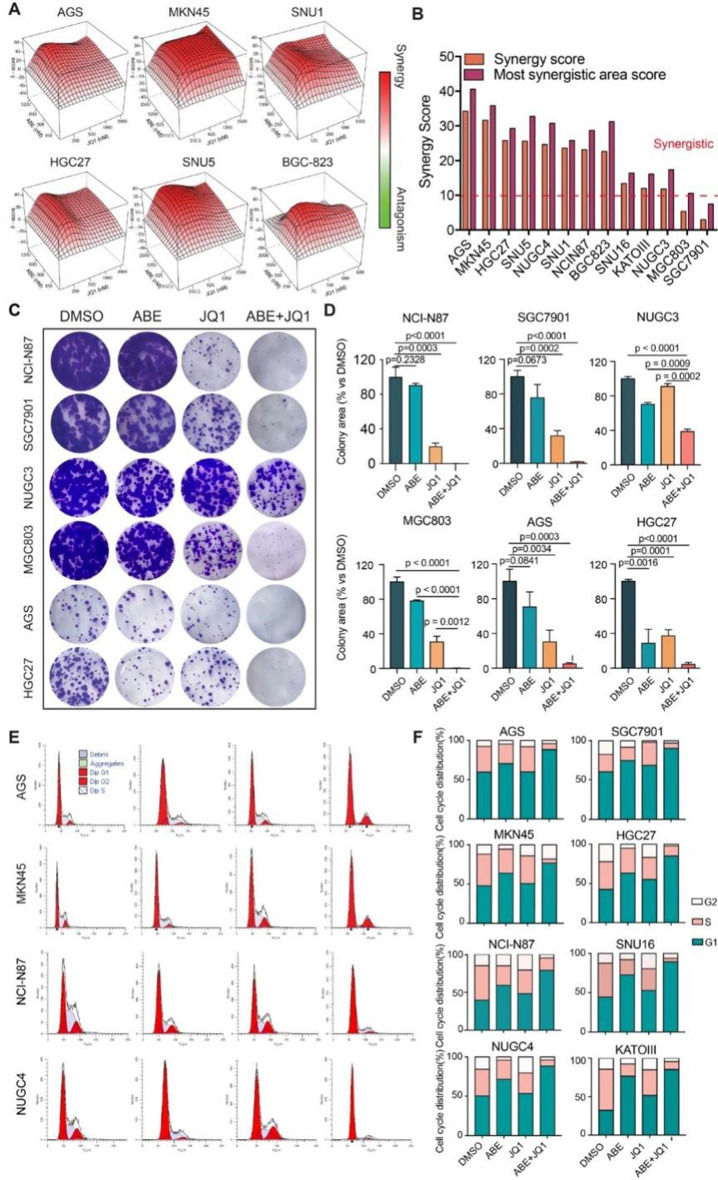
Cellular effects of the combination of ABE and JQ1 on in vitro GC cell models. **A** Representative synergy models of ABE and JQ1 across GC cell lines. **B** Bar plot of the average and maximum of synergy score among 13 GC cell lines. score > 10 indicates synergy. score < -10 indicates antagonism. **C** Crystal violet staining of colonies from six representative cell lines during 2 weeks with the indicated treatment of DMSO, ABE (100nM), JQ1(100nM), and ABE (100nM) + JQ1 (nM). **D** Quantification of the colonies area using imageJ software. The data are presented as the mean ± SEM of three replicates. **E** Representative Cell cycle plots of different GC cell lines treated with DMSO, ABE (1000nM), JQ1(500nM), or ABE (1000nM) + JQ1(500nM) for 48 h as examined by flow cytometry analyses. **F** Representative histograms of the ratio of G1, S, and G2 phase of GC cell lines with different treatments
