# Supplementary material for: The BRD4 inhibitor JQ1 augments the antitumor efficacy of abemaciclib in preclinical models of gastric carcinoma
Source: J Exp Clin Cancer Res. 2023 Feb 9;42:44. doi: 10.1186/s13046-023-02615-2 (PMC9909925; doi:10.1186/s13046-023-02615-2)
Supplement: Supplementary file 1 — Additional file 1: Figure S1. Pharmaceutical drug screening and Depmap sgRNA screening indicated that epigenetic inhibitor JQ1 and cell cycle inhibition are more likely to be associated with joint lethality. A) Bar plot of the average and maximum synergy score of abemaciclib combination with 6 epigenetic drugs in 2 GC cell lines. B) Rank plot showing the essential gene of cell-cycle related genes including CDK4, CDK6, CDK2, CCND1 and epigenetic regulator BRD4 in 35 gastric cancer cell lines form depmap dataset. Figure S2. Synergistic analysis of ABE and JQ1 for the in vitro tests. A) Dose-response curves for JQ1 in 13 GC cell lines. JQ1 was evaluated in 10 serially diluted doses and each dose was analyzed in technical quadruplicate in each biological replication. The relative cell number was determined using the CellTiter-GLo signal captured on the luminescent microplate reader. B) Average IC50 values generated from dose-response curves for JQ1. C) Representative synergy models of ABE and JQ1 across the indicted GC cell lines. D) Cell cycle of different GC cell lines treated with DMSO, ABE, JQ1, or ABE+JQ1 for 48h examined by flow cytometry analyses. Figure S3. The transcriptome profiling analysis of the four cohort treatments of AGS cells. A) Volcano plot showing the up and down regulated genes in the treatment of indicted agents in 12h and 24h, respectively. B) Venn diagram showing differentially expressed genes（DEGs）(q value < 0.05) by abemaciclib, JQ1, or combination compared with control DMSO in 12h and 24h respectively. C -D) The up and down REACTOME enrichment pathways in tumors upon the treatment of abemaciclib combination of JQ1 versus control DMSO treatment (24h). E) Expression levels of cell senescence, cell cycle checkpoint and DNA repair pathway signature genes for the 12h and 24 h treatment of abemaciclib, JQ1 and the combination of abemaciclib and JQ1 in AGS cell line. F-H) GSEA revealed that Homologous Recombination, E2F targets and Cell Senescence g [file 13046_2023_2615_MOESM1_ESM.docx]

Supporting information

The *BRD4* inhibitor JQ1 augments the antitumor efficacy of Abemaciclib in preclinical models of gastric carcinoma

*Mei Feng^1#^, Hao Xu^1#^, Wenyuan Zhou^2^, Yisheng Pan^1*^*


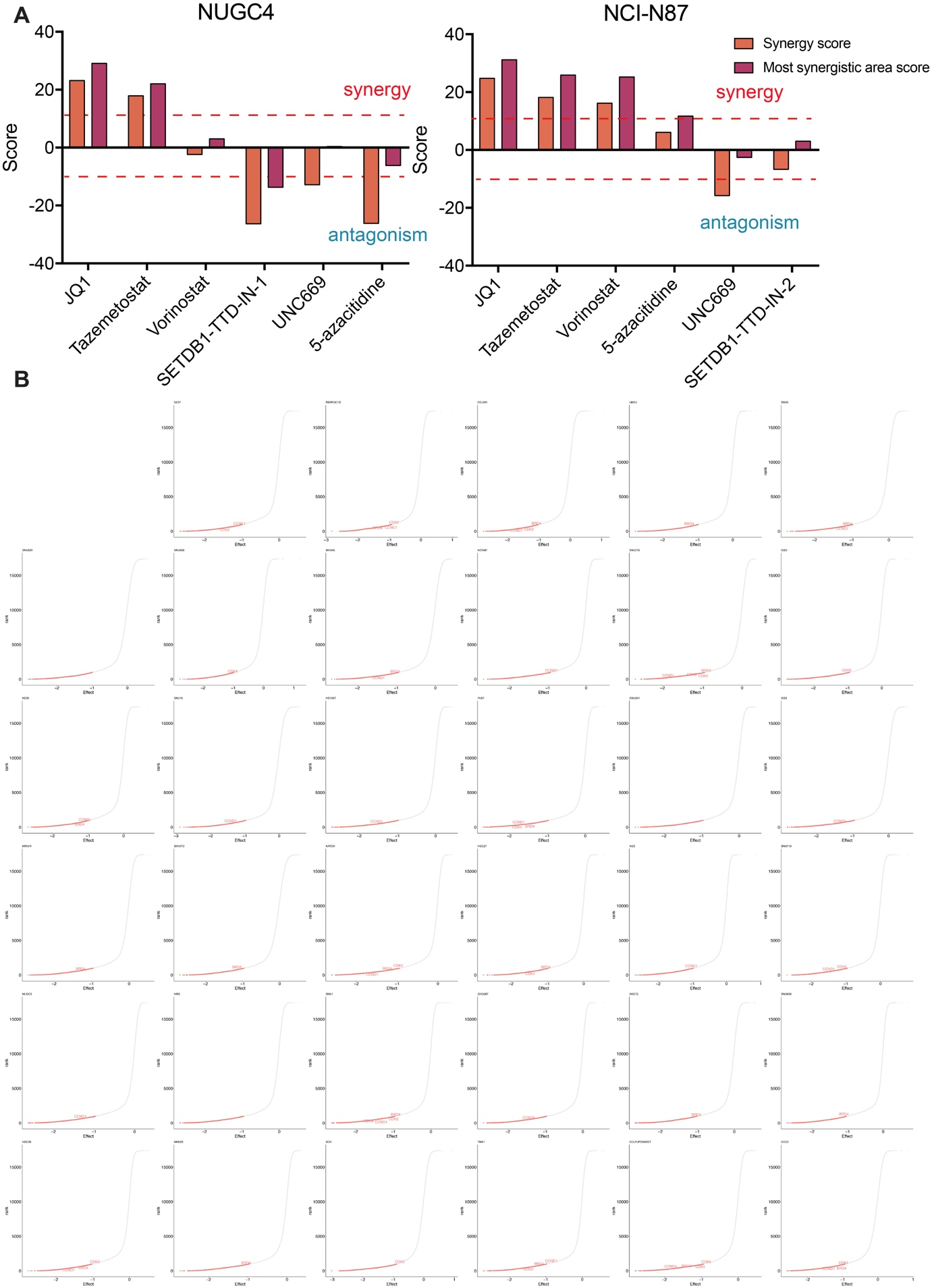


**Figure S1.** Pharmaceutical drug screening and Depmap sgRNA screening indicated that epigenetic inhibitor JQ1 and cell cycle inhibition are more likely to be associated with joint lethality. A) Bar plot of the average and maximum synergy score of abemaciclib combination with 6 epigenetic drugs in 2 GC cell lines. B) Rank plot showing the essential gene of cell-cycle related genes including CDK4, CDK6, CDK2, CCND1 and epigenetic regulator BRD4 in 35 gastric cancer cell lines form depmap dataset.


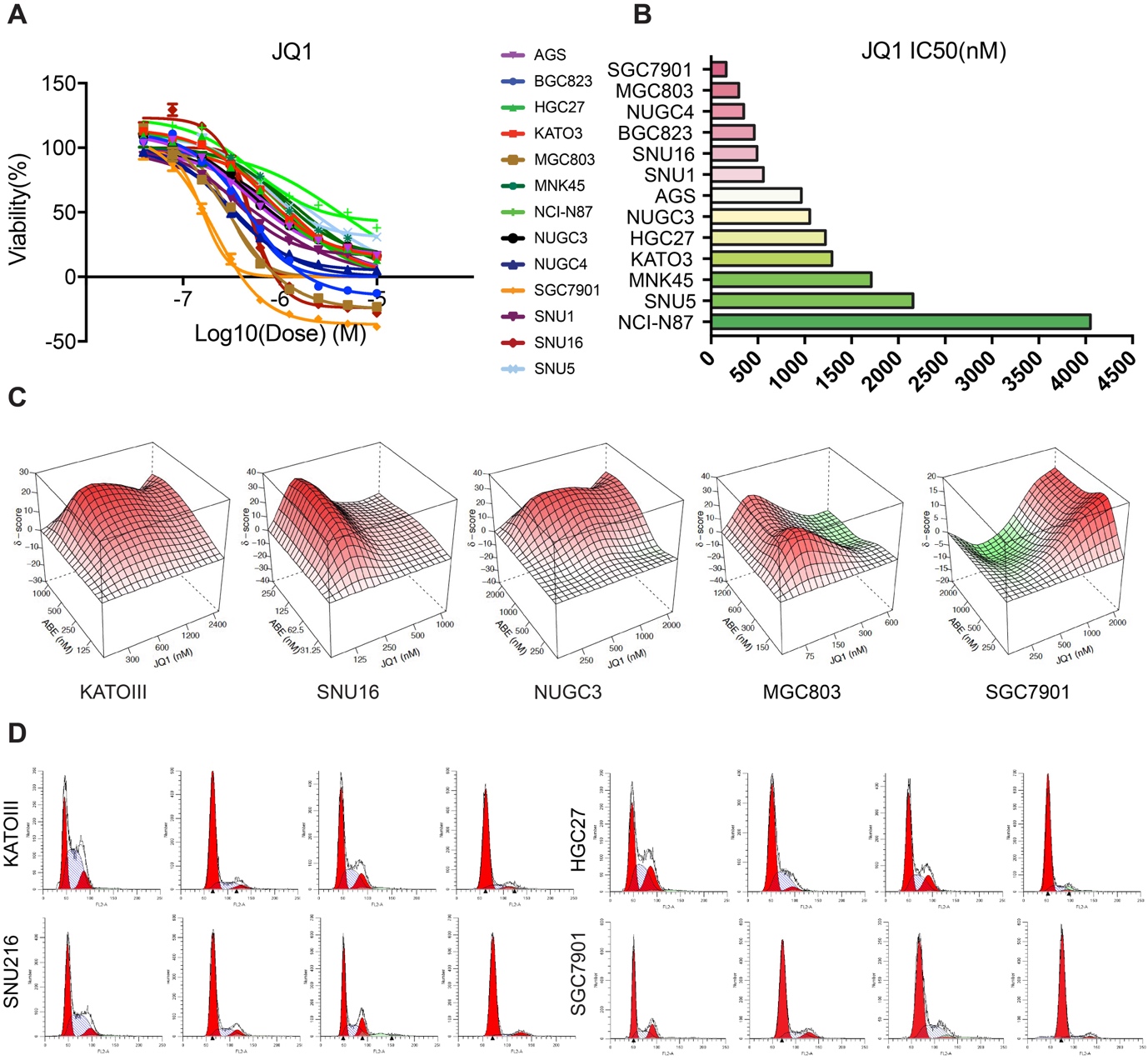


**Figure S2.** Synergistic analysis of ABE and JQ1 for the in vitro tests. A) Dose-response curves for JQ1 in 13 GC cell lines. JQ1was evaluated in 10 serially diluted doses and each dose was analyzed in technical quadruplicate in each biological replication. The relative cell number was determined using the CellTiter-GLo signal captured on the luminescent microplate reader. B) Average IC50 values generated from dose-response curves for JQ1. C) Representative synergy models of ABE and JQ1 across the indicted GC cell lines. D) Cell cycle of different GC cell lines treated with DMSO, ABE, JQ1, or ABE+JQ1 for 48h examined by flow cytometry analyses.


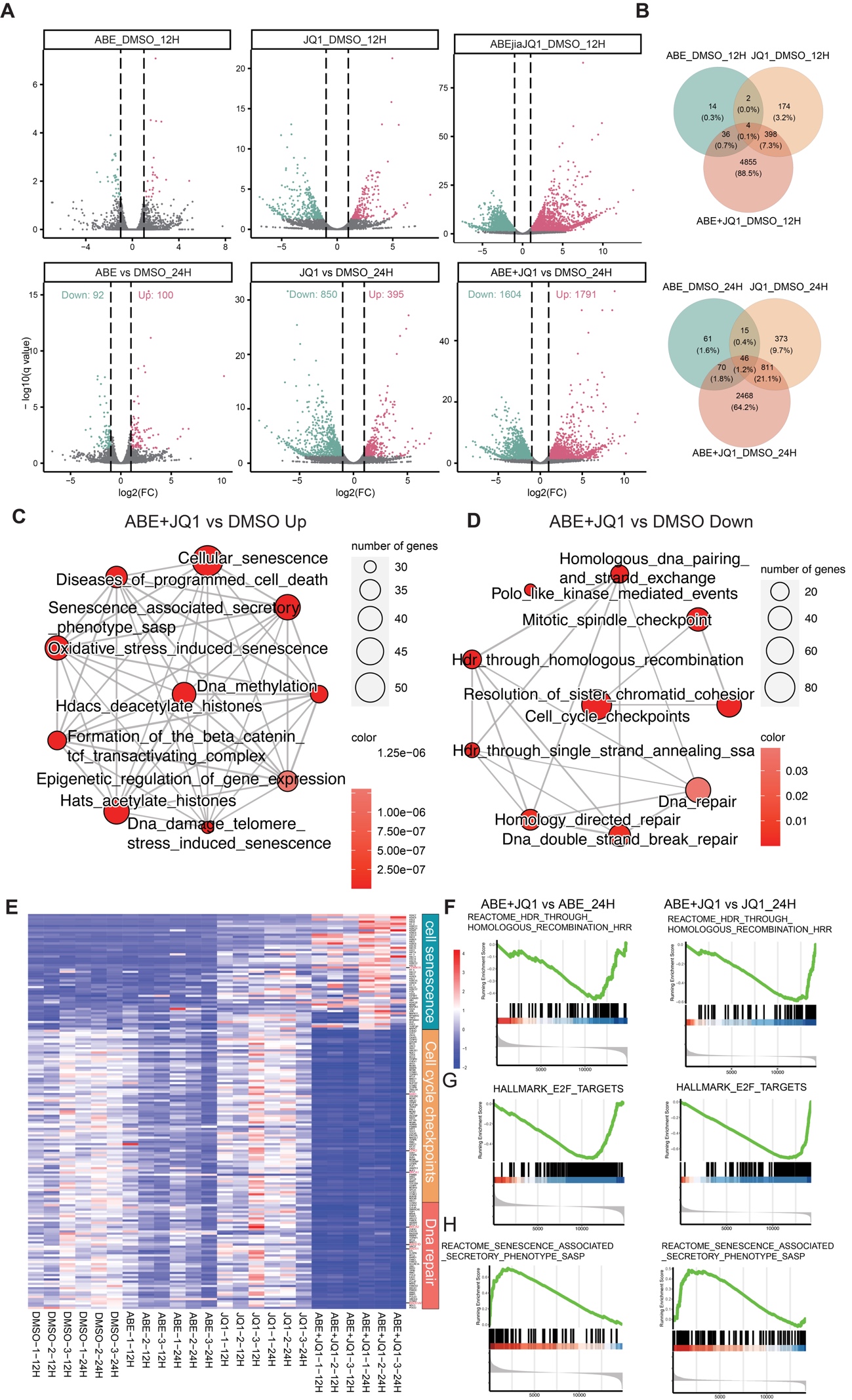


**Figure S3.** The transcriptome profiling analysis of the four cohort treatments of AGS cells.

A) Volcano plot showing the up and down regulated genes in the treatment of indicted agents in 12h and 24h, respectively. B) Venn diagram showing differentially expressed genes（DEGs）(q value < 0.05) by abemaciclib, JQ1, or combination compared with control DMSO in 12h and 24h respectively.

C -D) The up and down REACTOME enrichment pathways in tumors upon the treatment of abemaciclib combination of JQ1 versus control DMSO treatment (24h).

E) Expression levels of cell senescence, cell cycle checkpoint and DNA repair pathway signature genes for the 12h and 24 h treatment of abemaciclib, JQ1 and the combination of abemaciclib and JQ1 in AGS cell line.

F-H) GSEA revealed that Homologous Recombination, E2F targets and Cell Senescence gene sets are enriched in ABE+JQ1-treated AGS cells using RNA sequencing data.


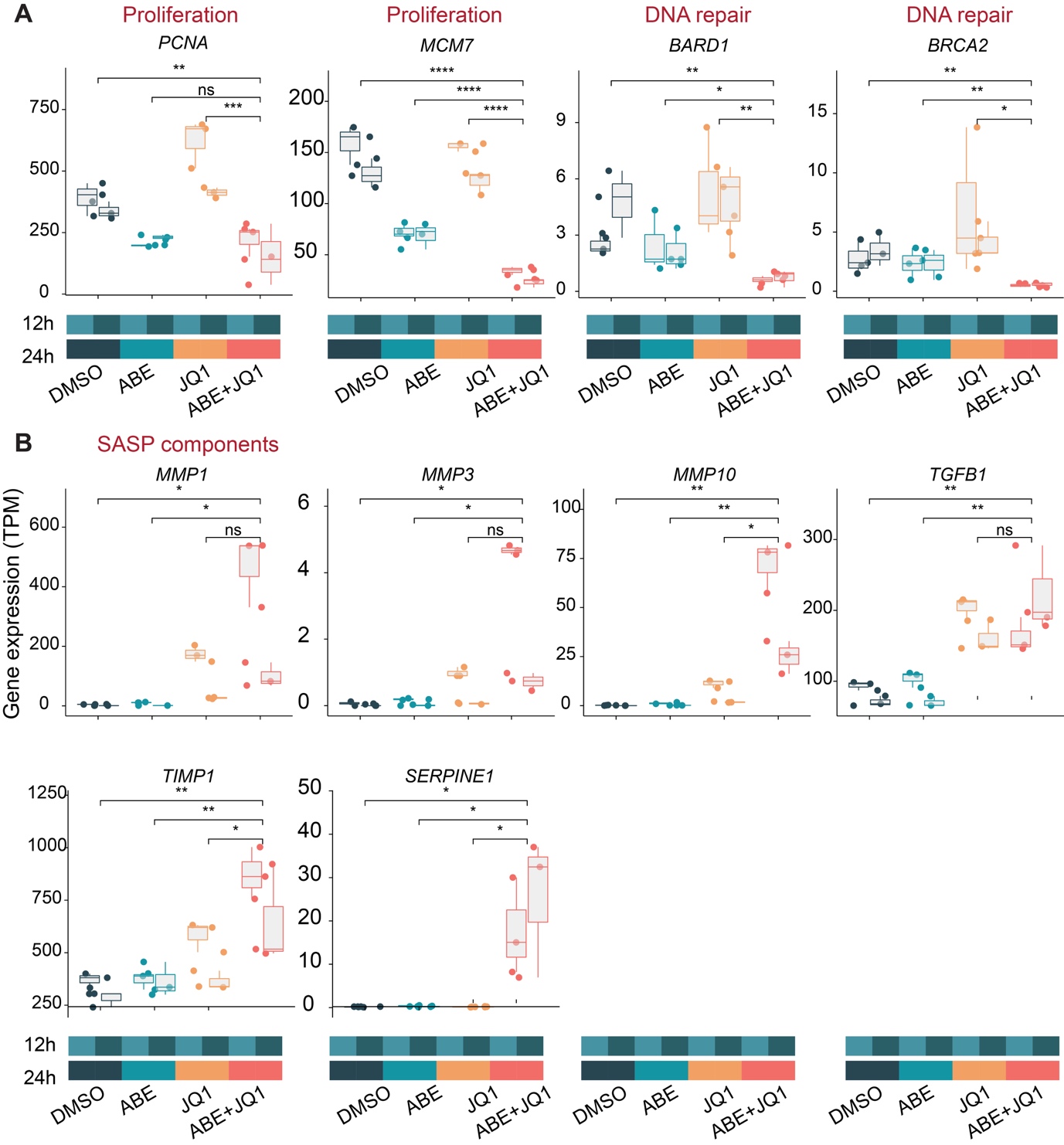


**Figure S4.** The transcriptome profiling analysis demonstrated the downregulation of proliferation and DNA repair genes and the upregulation of SASP genes in the ABE+JQ1 combination cohort. A) Box plots showing the expression levels of marker genes of PROLIFERATION (*PCNA, MCM7*) and DNA REPAIR (*BARD1*, *BRCA2*). B) Box plots showing the expression levels of marker genes of SASP (*MMP1, MMP3, MMP10, TGFB1, TIMP1, SERPINE1).*

Table S1. Panel of 13 gastric cancer cell lines used in the study

| **Name** | **Median** | **sex** | **source** | **collection_site** | **primary_or_metastasis** |
| --- | --- | --- | --- | --- | --- |
| SNU5 | IMDM + 20% FBS | Female | ATCC | ascites | Metastasis |
| MKN45 | RPMI + 10% FBS | Female | CSIC | liver | Metastasis |
| NCIN87 | RPMI + 10% FBS | Male | ATCC | liver | Metastasis |
| SNU16 | RPMI + 10% FBS | Female | ATCC | ascites | Metastasis |
| NUGC4 | RPMI + 10% FBS | Female | RIKEN | lymph_node | Metastasis |
| KATOIII | IMDM + 20% FBS | Male | ATCC | pleural_effusion | Metastasis |
| HGC27 | RPMI + 10% FBS | Female | ECACC | lymph_node | Metastasis |
| AGS | RPMI + 10% FBS | Female | ATCC | stomach | Primary |
| NUGC3 | RPMI + 10% FBS | Male | CSIC | soft_tissue | Metastasis |
| SNU1 | RPMI + 10% FBS | Male | ATCC | stomach | Primary |
| MGC803 | DMEM + 10% FBS | Male | CSIC | stomach | Primary |
| BGC823 | DMEM + 10% FBS | Male | CSIC | stomach | Primary |
| SGC7901 | RPMI + 10% FBS | Female | CSIC | lymph_node | Metastasis |

Table S2. In vitro drug dose response for monotherapy abemaciclib and JQ1 in Gastric cell lines.

| Cell line | Abemaciclib (nM) | JQ1(nM) |
| --- | --- | --- |
| SNU16 | 126.2 | 490 |
| MNK45 | 194.7 | 1712 |
| SNU1 | 455.9 | 557.9 |
| NUGC4 | 534 | 349.9 |
| KATO3 | 639.3 | 1292 |
| AGS | 685.2 | 964.5 |
| HGC27 | 718.8 | 1222 |
| SNU5 | 842.9 | 2157 |
| MGC803 | 948.8 | 297.3 |
| NUGC3 | 1034 | 1053 |
| BGC823 | 1271 | 460.1 |
| NCI-N87 | 1574 | 4051 |
| SGC7901 | 2104 | 160.1 |

Table S3. Real-time pcr primers of p21, p53 and 53bp1 used in this study

| Primer | Sequencing |
| --- | --- |
| p21-qpcr-F | AATTGGAGTCAGGCGCAGAT |
| p21-qpcr-R | CGGACATCACCAGGATTGG |
| Trp53-qpcr-F | CACCCTGGCTAAAGTTCTGT |
| Trp53-qpcr-R | CATCCAGTCTTCGGAGAAGC |
| 53bp1-qpcr-F | CAGCAGAGTTTGTGAGTCCC |
| 53bp1-qpcr-R | GTTGTAGCCATGGTGAGGAG |
